# Self-resistance in *Streptomyces*, with Special Reference to β-Lactam Antibiotics

**DOI:** 10.3390/molecules21050605

**Published:** 2016-05-10

**Authors:** Hiroshi Ogawara

**Affiliations:** 1 HO Bio Institute, 33-9, Yushima-2, Bunkyo-ku, Tokyo 113-0034, Japan; hogawara@sc5.so-net.ne.jp; 2 Department of Biochemistry, Meiji Pharmaceutical University, 522-1, Noshio-2, Kiyose, Tokyo 204-8588, Japan

**Keywords:** β-lactam antibiotics, β-lactamase, penicillin-binding protein, self-resistance, antibiotic resistance, *Streptomyces*, *Actinobacteria*

## Abstract

Antibiotic resistance is one of the most serious public health problems. Among bacterial resistance, β-lactam antibiotic resistance is the most prevailing and threatening area. Antibiotic resistance is thought to originate in antibiotic-producing bacteria such as *Streptomyces*. In this review, β-lactamases and penicillin-binding proteins (PBPs) in *Streptomyces* are explored mainly by phylogenetic analyses from the viewpoint of self-resistance. Although PBPs are more important than β-lactamases in self-resistance, phylogenetically diverse β-lactamases exist in *Streptomyces*. While class A β-lactamases are mostly detected in their enzyme activity, over two to five times more classes B and C β-lactamase genes are identified at the whole genomic level. These genes can subsequently be transferred to pathogenic bacteria. As for PBPs, two pairs of low affinity PBPs protect *Streptomyces* from the attack of self-producing and other environmental β-lactam antibiotics. PBPs with PASTA domains are detectable only in class A PBPs in *Actinobacteria* with the exception of *Streptomyces*. None of the *Streptomyces* has PBPs with PASTA domains. However, one of class B PBPs without PASTA domain and a serine/threonine protein kinase with four PASTA domains are located in adjacent positions in most *Streptomyces*. These class B type PBPs are involved in the spore wall synthesizing complex and probably in self-resistance. Lastly, this paper emphasizes that the resistance mechanisms in *Streptomyces* are very hard to deal with, despite great efforts in finding new antibiotics.

## 1. Introduction

The first antibiotic, penicillin, was discovered in 1929 by Fleming [1] and rediscovered in 1940 by Chain *et al.* [2] and in 1941 by Abraham *et al.* [3]. Surprisingly, even before the rediscovery of penicillin, a bacterial penicillinase (β-lactamase) was reported [4] and only few years later, penicillin-resistant *Staphylococcus aureus* [5,6] and *Streptococcus pneumoniae* [7] were already described, suggesting strongly that the resistant trait was intrinsically present in their genomes of these bacteria and, in addition, penicillin-inactivating substances (β-lactamases?) were speculated to be involved in the resistance [8]. Gram-negative bacteria such as *Neisseria gonorrhoeae* [9], *Neisseria meningitides* [10] and *Escherichia coli* [11] were intrinsically resistant. The same phenomenon was also observed with streptomycin [12]. That the resistance traits were inherently present in the genomes of *Streptomyces* was supported by the fact that the percentage production of β-lactamases in newly isolated *Streptomyces* strains and those isolated thirty years ago from soil was not so different with each other at the time in 1978 [13]. If the selective pressure of the use of β-lactam antibiotics would affect the resistant trait, the relative frequencies of β-lactamase producing *Streptomyces* strains newly isolated from soil would be much more, indicating that the selective pressure at that time is not so strong as at the present time. Recently, however, D’Costa *et al.* demonstrated that genes conferring resistance to tetracyclines, vancomycin, aminoglycosides, β-lactams and macrolides existed in 30,000-year old Beringian permafrost and that the amino acid sequences of the β-lactamases showed identities between 53% and 84% with those of known determinants, suggesting that antibiotic resistance is a natural phenomenon that predates the modern selective pressure of clinical antibiotic use [14]. 

Drug resistance is one of the greatest challenges in modern medicine. Among drug resistance, antibiotic resistance is the most prevailing and threatening topic in public health. According to the information of the Centers for Disease Control and Prevention (CDC), at least 2 million people become infected with bacteria that are resistant to antibiotics and at least 23,000 people die each year as a direct result of these infections in the United States each year [15], and in 2009 the Antibiotic Resistance Genes Database (ARDB) contained resistance information for 13,293 genes [16]. These resistance genes are hypothesized to originate in the antibiotic-producing bacteria such as *Streptomyces* [17,18,19]. Surprisingly, however, when Dantas *et al.* cultured clonal isolates from 11 different soils that could use one of 18 different antibiotics as their sole carbon source, phylogenetic profiling of the clonal isolates revealed a diverse set of species consisting of orders *Burkholderiales* (41%), *Pseudomonadales* (24%) and *Actinomycetales* (7%) and others and, indicated that pathogenic microbes can more readily use resistance genes originating from bacteria subsisting on antibiotics than the resistance gene from more distantly related antibiotic producer bacteria [20]. *Burkholderiales* and *Pseudomonadales* belong to the proteobacteria and are known to function as scavengers, capable of using a large variety of single carbon sources as food. However, horizontal gene transfer is possible from proteobacteria such as *Burkholderiales* and *Pseudomonadales* to *Actinomycetales* including antibiotic-producing *Streptomyces* in soil and then to pathogenic bacteria. From the result of a metagenomic analysis to isolate antibiotic resistance genes from soil, Riesenfeld *et al.* concluded that soil bacteria are a reservoir of antibiotic resistance genes with greater diversity than previously recognized [21]. However, D’Costa *et al.* described that soil dwelling bacteria produce and encounter a myriad of antibiotics and are a reservoir of resistance determinants (resistome) [22], although they used strains in soil samples collected from diverse locations such as urban, agricultural field, and forest but resembled actinomycetes both morphologically and microscopically [23]. Using multidrug-resistant proteobacteria from the soil, Forsberg *et al.* isolated 110 resistance genes. Of the 110 resistance genes, 18 had 100% amino acid identity to entries in GenBank and another 32 were highly similar. Interestingly, of 110 resistance genes, 55 were β-lactamase genes highly divergent from those of the antibiotic-producing *Streptomyces*. From these results they indicated ancient evolutionary relationships between β-lactamases from the soil bacteria and those of the antibiotic-producing bacteria [24]. However, β-lactamases from proteobacteria belong mainly to class C β-lactamases, while those from *Streptomyces* are class A β-lactamases [19]. So, it is not surprising that these two β-lactamases are only distantly related as described below. In any way, it is more reasonable to speculate that the resistance genes originated in antibiotic producing microorganisms and these resistance genes were developed from natural sources to clinic, human, animal, and other environments by transduction, transformation and conjugation to make the enormous diversity of antibiotic resistance genes and no barriers among the microbial compartments in the microbial world at the present time [25,26,27,28,29].

The phylum *Actinobacteria* constitute one of the largest phyla within the bacteria [30,31]. *Streptomyces* species belonging to the *Actinobacteria* dwell in the soil and are high guanine + cytosine (G + C)-content Gram positive bacteria. They are characterized by their peculiar morphology to undergo complex cellular differentiation like filamentous fungi [32] and by their production of a wide variety of secondary metabolites including β-lactam antibiotics [33,34] such as thienamycin [35] and clavulanic acid [36]. However, unlike penicillin- and cephalosporin-producing fungi, *Streptomyces* species are prokaryotic microorganisms so they must protect themselves from the attack of antibiotics to avoid suicide, that is, they have to have self-resistance mechanisms. In addition, β-lactam antibiotics have been most widely used for chemotherapy of infectious diseases even about 90 years after penicillin’s discovery, even though the chemical structures were extensively modified. β-Lactam antibiotic resistance is caused mainly by two mechanisms: antibiotic-degrading enzymes, β-lactamases and modification of target sites, penicillin-binding proteins (PBPs). *Streptomyces* species are known to be highly resistant to benzyl penicillin, although they are Gram-positive bacteria, so it is interesting to know the self-resistance mechanisms in *Streptomyces* and the relationships between PBPs, β-lactams and β-lactamases [19,37]. This review discusses the self-resistance in *Streptomyces*, focusing primarily on β-lactam antibiotics. 

## 2. Self-resistance mechanisms

As described above, the self-resistance in antibiotic-producing bacteria such as *Streptomyces* is at least one of the origins of multi-drug resistance prevailing and threatening in various environments at the present time. Major biochemical mechanisms of drug resistance are detoxication or inactivation of the drug, changing the target site, blocking the transport of the drug into the cell and the efflux pump system [19,38]. These mechanisms are true with the self-resistance mechanisms or, bacteria use very similar or the same mechanisms for self-resistance in the antibiotic-producing bacteria and antibiotic resistance in pathogenic bacteria. While the major resistance mechanism in pathogenic bacteria is β-lactamases, that in *Streptomyces* is supposed to be penicillin-binding proteins [19]. A number of excellent review papers and databases have been published on antibacterial multidrug resistance and self-resistance [19,39,40,41,42,43].

### 2.1. β-Lactamases

β-Lactamases are the enzymes which catalyze the hydrolysis of the β-lactam antibiotics to produce antimicrobiologically inactive compounds. Because of this, β-lactamases are responsible for β-lactam resistance in many pathogenic bacteria [44]. These enzymes, however, are also produced by nonpathogenic bacteria such as *Streptomyces* [13,45], *Bacillus* [46] and the cyanobacteria [47,48]. In addition, recent whole genome analyses expand the β-lactamase world to β-lactamase superfamily proteins [49,50], β-lactamase domain-containing proteins [51] and RNA-metabolizing metallo-β-lactamases [52,53]. Consequently, β-lactamase-producing organisms include a thermobacterium (*Meiothermus ruber* DSM 1279) (GenBank accession number, ADD27888, the same hereafter, class C β-lactamase), a green sulfur bacterium (*Pelodictyon phaeoclathratiforme* BU-1, ACF43212, a metallo-β-lactamase), a green non-sulfur bacterium (*Herpetosiphon aurantiacus* DSM 785, ABX07048, a metallo-β-lactamase), a methylotrophic bacterium [54] (*Methylovorus glucosetrophus* SIP3-4, ACT50532, a metallo-β-lactamase), a fungus (*Schizosaccharomyces pombe*, CAA93219, a tRNA processing endonuclease, a metallo-β-lactamase) and even a plant [55] (*Arabidopsis thaliana*, AAC49866, a glyoxalase II mitochondrial enzyme) and mammals [56,57] (*Rattus norvegicus* and *Homo sapiens*, EDL84248 “a serine β-lactamase-like protein” and NP_116246 “a serine β-lactamase-like mitochondria protein”, respectively). Furthermore, an alkalophilic and halotolerant Gram-positive bacterium *Oceanobacillus iheyensis* isolated from the bottom of the Pacific Ocean at a depth of 1050 m [58] and *Pseudomonas fluorescens* isolated from a remote Antarctic coastal area [59] also produce β-lactamases. Moreover, screening of metagenomic libraries from soil bacterial communities for clones that confer antibiotic resistance gave new types of β-lactamases such as oligomeric [60] and bifunctional β-lactamases [61,62]. A bifunctional β-lactamase was also isolated from a pathogen *Mycobacterium tuberculosis* H37Rv, an *Actinobacterium* [63,64]. Therefore, various kinds of β-lactamases or β-lactamase superfamily are distributed in a variety of organisms, but in this review β-lactamases are described in a more classical sense. 

β-Lactamases are grouped into four classes on the basis of their amino acid sequences [65,66,67,68,69,70,71,72]. This classification reflects more or less the substrate specificity: class A (penicillin-hydrolyzing), class C (cephalosporin-hydrolyzing) and class D (oxacillin-hydrolyzing) β-lactamases are active site serine enzymes and class B β-lactamases require one or two zinc ions for their activity and are called as metallo-β-lactamases [69,73,74,75,76]. A previous phylogenetic study indicated that class A β-lactamases could be divided into three subgroups [77]. β-Lactamases are not essential bacterial proteins in themselves, but are speculated to have evolved from the essential PBPs in some β-lactam-producing bacteria such as *Streptomyces* or related bacteria, because these bacteria have to have some self-protective strategies against β-lactam antibiotics [19,48]. PBPs are involved in peptidoglycan biosynthesis in prokaryotic microorganisms. Therefore, it is interesting to know the relationships of β-lactam antibiotics, β-lactamases and PBPs in *Streptomyces*, β-lactam-producing microorganisms. In addition, the relationship of β-lactamases in *Streptomyces* and those in pathogenic bacteria should be clarified. In this section, the phylogenetic relationships were analyzed among β-lactamases in *Streptomyces* and a couple of pathogenic bacteria, whose genome sequences were wholly clarified. 

Previously, I investigated the characteristics of 59 β-lactamase candidate sequences among 529 putative β-lactamase and PBP sequences in 19 *Actinobacteria* [78,79], which were collected from the NCBI database [80]. There were some characteristic features in the 59 candidate sequences. First, β-lactamase candidates were not distributed uniformly in the strains: while *Saccharomonospora viridis*, *Streptomyces avermitilis*, *Streptomyces scabiei* and *Streptosporangium roseum* possess as many as five, twelve, five and five candidate genes, respectively, *Mycobacterium leprae*, *Nocardioides* sp. JS614 and *Rhodococcus erythropolis* had no candidate genes. In general, *Streptomyces* species produced β-lactamase candidates at higher rates. However, the numbers of candidate sequences were not necessarily related to the taxonomic classification. For example, although *Streptosporangium* and *Nocardioides* were closely related taxonomically [81], the numbers of β-lactamase candidates in *Streptosporangium* were five but those in *Nocardioides* were zero. Even in the same genus, *M*. *tuberculosis* produced four but *M. leprae* produced none. β-Lactamases detected by their enzymatic activity also distributed independently of actinobacterial taxonomy [82]. These results indicate strongly that β-lactamases evolve and are produced independent of taxonomy.

Second, most of the β-lactamases detected so far by their enzymatic activity in Gram-positive bacteria belong to class A and a few belong to class B. However, this fact was not the case with whole genomic level.

Third, it is intriguing that *Thermomonospora curvata*, a high G + C Gram-positive soil thermophilic *Actinobacteria*, produced two class B and one class D β-lactamase candidates but no class A and class C enzymes. Usually, Gram-positive bacteria produce class A and class B β-lactamases, but not class C and class D. In addition, *T. curvata* DSM43183 Tcur2040 β-lactamase is closely related to Gram-negative class D β-lactamases, especially OXA-5 and OXA-27 type β-lactamase [79], although *T. curvata* is a Gram positive bacteria. Hereafter, genetic analyses are extended to β-lactamases of other 12 *Streptomyces* species listed in Table 1.

#### 2.1.1. Class A β-lactamases

The amino acid sequences of putative 25 class A β-lactamases together with a reference sequence (TEM-1, *Klebsiella penumoniae* plasmid, YP_005351445) were aligned by using the Muscle and T-coffee programs [83,84]. The results showed that all the sequences in Table 2 possessed main residues involved in the catalytic reaction (Table 3), except SCAT_1418, SCAT_4581, SRIM_07318, and F750_2387 (Table S1). These four sequences are defective in S^70^XXK^73^ and S^130^DN^132^. Furthermore, SCAT_1418, SCAT_4581 and F750_2387 do not carry K^234^T/SG^236^, indicating that these candidates do not function as β-lactamases (Table 2).

Although some other sequences are also deficient or possess incompletely identical signature amino acid sequences, they are supposed to operate as β-lactamases even if the catalytic efficiency is not perfect. GWG^166^ in SSCG_00160, GWG^166^ in F750_6336, IWE^166^ in B446_02285, IWE^166^ in B466_33010, RVI^166^ in STSU_15177, RGI^166^ in SCAT_0807, HYE^166^ in SCAT_5692, QYE^166^ in SSCG_00130, and RWS^166^ in F750_2387 are examples. In fact, blast analysis with SSCG_00160 as a query, putative β-lactamases from *Saccharomonospora viridis* (KHF44103), *Amycolicicoccus subflavus* DQS3-9A1 (AEF33802) and *Rhodococcus* sp. B7740 (AJW42765) were identified.

In addition blast analysis using SCAT_1418 as a query, metallo-dependent hydrolase of *Streptomyces violaceusniger* (WP_014060235) and metallo-dependent hydrolase of *Streptomyces himastatinicus* (WP_00971837) were identified. Actually, SCAT_1418 possesses some class B β-lactamase signature residues. Likewise, SCAT_4581 is highly similar to Zn-dependent hydrolases from *Actinobacteria*, so that they may be class B β-lactamases.

SRIM_07318 is too short to function as a β-lactamase, but may be a part of class A β-lactamase, as its amino acid sequence of the C-terminal part is very similar to that of class A β-lactamases from *Streptomyces albus* (WP_060729068) and *Rhodococcus rhodnii* (WP_037260801). F750_2387 is a large membrane protein and belongs to metallo-β-lactamase superfamily. The fact that SCAT_1418, SCAT_4581 and F750_2387 belong to metallo-β-lactamases but not to class A reflects on large molecular distances between these β-lactamases and others such as TEM-1 and SHV-1 (Table S2). Interestingly, the amino acid sequence of SRIM_10531 is completely identical with that of *Streptomyces cellulosae* (D12653). This is one example of the horizontal gene transfer. Another interesting thing is that the amino acid sequences of B446_02285 and B446_33010 in class A β-lactamases, and B446_02210 and B446_33085, and B446_02000 and B446_33290 in class B β-lactamases, and B446_01720 and B446_33570 in β-lactamase-like sequences and B446_01315 and B446_33975 in class C β-lactamases are also completely identical, indicating that about 7.7 Mb region including these genes were duplicated in the chromosome in the past. In fact, two transposases exist at both sides in reverse direction from 7,984,885-7,985,573n and 287,352-288,040n in *Streptomyces collinus* genome, respectively, suggesting that these transposases acted in the transposition or the duplication. 

**Figure 1:**
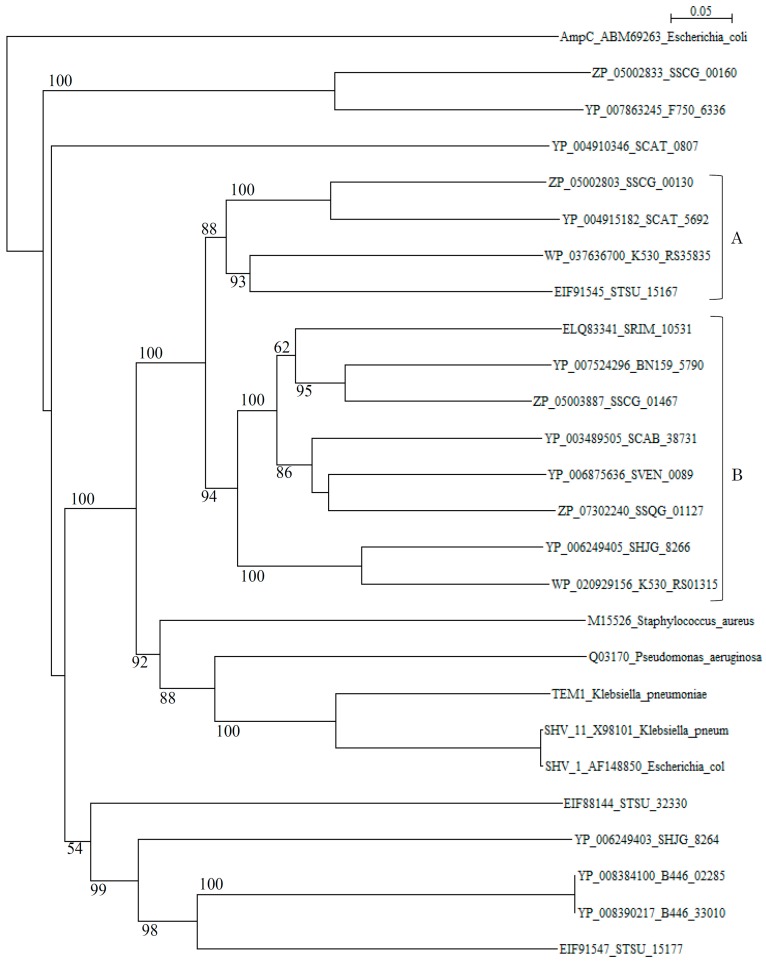
Phylogenetic tree of putative 20 class A β-lactamases on the basis of amino acid sequences with those of *Staphylococcus aureus* (M15526), *Pseudomonas* *aeruginosa* (Q03170), *Klebsiella pneumoniae* (TEM-1, YP_005351445), *Klebsiella pneumoniae* (SHV-11, X98101), and *Escherichia* *coli* (SHV-1, AF148850) as reference sequences. The tree was constructed by using ClustalX 2 [88] and *E. coli* AmpC (ABM69263) as outgroup. Clusters A and B show blue-dextran/NADP^+^-non-binding and binding β-lactamases from *Streptomyces*, respectively and, cluster C shows those from pathogenic bacteria used as reference sequences. The bootstrap probabilities are shown at branching nodes.

A phylogenetic tree of putative 20 class A β-lactamases constructed with those of *S. aureus* (M15526), *P*. *aeruginosa* (Q03170), *K. pneumoniae* (TEM-1, YP_005351445), *K. pneumoniae* (SHV-11, X98101), and *E. coli* (SHV-1, AF148850) as reference sequences disclosed that although these reference sequences are originated from quite different species, they form only one cluster (cluster C in Figure 1). In contrast, β-lactamases of *Streptomyces* are scattered in various branches, indicating that only a few β-lactamase genes of *Streptomyces* species have been transferred to pathogenic bacteria at the present time.

However, it is possible that other β-lactamase genes of *Streptomyces* species may transfer to pathogenic bacteria in the near future, although many threatening class A genes may be evolved through mutation but not transferred to pathogenic bacteria, as it is seen in TEM-type and SHV-type β-lactamases [89]. Actually, molecular distances determined by the protdist program [90] between most class A β-lactamases of *Streptomyces* and those of pathogenic bacteria (Table S2) are not so large as in class B and class C β-lactamases, indicating that these class A β-lactamases are related closely with each other and with those of pathogenic bacteria than class B and class C enzymes. It is speculated, therefore, that the horizontal gene transfer of class A β-lactamase genes is easier than for class B and class C β-lactamases. The β-lactamases of *Streptomyces* can be classified into blue-dextran/NADP^+^-binding and non-binding groups [91,92]. Blue-dextran/NADP^+^-non-binding β-lactamases belong to cluster A and those of binding β-lactamases go to cluster B in the phylogenetic tree (Figure 1). However, the role of NADP+-binding of β-lactamases remains to be clarified. 

As for the relationship between class A β-lactamases and the β-lactam biosynthetic gene cluster, SSCG_00130 (corresponding to SCLAV_4216, partial stop and SCAT_5692) is located in the terminal region of the cephamycin biosynthetic gene cluster and SSCG_00160 (corresponding to SCLAV_4187) is located within the clavulanic acid biosynthetic gene cluster in *S. clavuligerus* (Figure S1). These two clusters exist in the adjacent position. SSCG_01467 (corresponding to SCLAV_2436) is not related to β-lactam biosynthetic genes [78]. Similarly, SCAT_5692 is present in the terminal region of the cephamycin biosynthetic gene cluster in *S. cattleya* (Figure S2). However, a gene corresponding to SSCG_00160 in *S. clavuligerus* is missing in *S. cattleya* in accord with the absence of clavulanic acid biosynthesis. Therefore, it is suggested strongly that class A β-lactamases and β-lactam biosynthesis are closely related with each other. However, it remains to be clarified whether these β-lactamases are involved in the self-resistance in the *Streptomyces* species. SSCG_00160 (SCLAV_4187) is also thought to be involved in clavulanic acid biosynthesis [93,94].

#### 2.1.2. Class B β-lactamases

On the basis of phylogenetic and sequences analyses, 63 class B β-lactamase candidates were selected in 12 *Streptomyces* species. The amino acid sequence alignment using the Muscle and T-coffee programs [83,84] revealed that all the sequences contain the conserved active site residues (HXHXD^120^) except SSCG_05130, SHJG_8335, SCAT_4145, and SRIM_16845 (Table 4 and Table S3; In the case of AAA22276_*Bacillus cereus* reference sequence, the active site residues are shifted to downstream in Table S3). 

**Figure 2:**
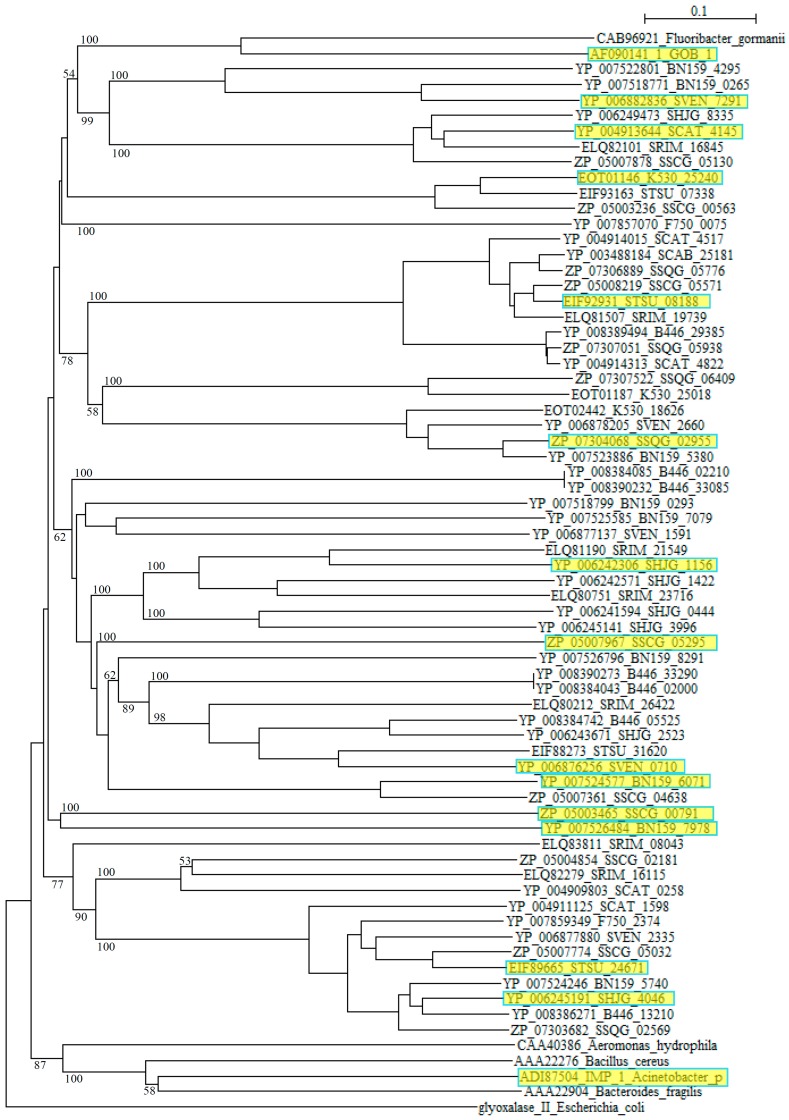
Phylogenetic tree of putative 63 class B β-lactamases on the basis of amino acid sequences. The tree was constructed by using ClustalX 2 [88] and *Escherichia coli* glyoxalase II (CDZ19109) as outgroup. β-Lactamases from *Fluoribacter gormanii* (CAB96921), *Elizabethkingia meningoseptica* GOB-1 (AF090141_1), *Aeromonas hydrophila* (CAA40386), *Bacillus cereus* (AAA22276), *Acinetobacter pittii* IMP_1 (ADI87504), and *Bacteroides fragilis* (AAA22904) were used as reference sequences. The sequences marked with yellow boxes were used for the calculation of molecular distances (Table S4). The bootstrap probabilities are shown at branching nodes.

However, blast analyses using these sequences as queries clarified that they are closely related to αββα metallo β-lactamase (MBL) fold hydrolases from *Streptomyces* such as *S. roseochrmogenus* (WP_023553317), *S. silaceus* (WP_055701146), *S. sclerotialus* (WP_030612498), *S. corchorusii* (WP_059262406), *S. hygroscopicus* (WP_058083348), *S. achromogenes* (WP_030611171), *S. bingchenggensis* (WP_014181511), *S. alboniger* (WP_055536123), *S. rimosus* (WP_033031918), *S. albus* (WP_060732298), *S. monomycini* (WP_030021421) and others, indicating that these sequences belong to the metallo β-lactamase superfamily. As in the case of class A, while the class B β-lactamases of pathogenic bacteria form compact clusters in a phylogenetic tree, those from *Streptomyces* are dispersed quite extensively (Figure 2). 

Moreover, comparing to Class A and class C β-lactamases, molecular distances calculated by using the protdist program [90] are mostly quite large between those of *Streptomyces* species with each other and those with pathogenic bacteria (Table S4), suggesting that the class B β-lactamase enzymes are assemblies of highly diverged proteins. Recently, new type of metallo-β-lactamases such as New Delhi metallo-β-lactamase (NDM) have emerged and swiftly spread worldwide. It is possible that various metallo-β-lactamases of *Streptomyces* are also transferred to pathogenic bacteria and pose a similar public health threat.

#### 2.1.3. Class C β-lactamases

The amino acid sequence alignment of putative 94 class C β-lactamases is shown in Table S5. From this alignment, the presence or absence of the active site residues was inferred and the results was shown in Table 5. All the sequences possess active site residues shown in Table 3, with exceptions of SHJG_8639 (YP_006249777), SHJG_2828 (YP_006243975), SSCG_03668 (ZP_05006222), SRIM_31085 (ELQ79325), BN159_7932 (YP_007526438), SSQG_02491 (ZP_07303604), and SSQG_00225 (ZP_07301338). However, the N-terminal sequences of SHJG_2828, SSCG_03668, SRIM_31085, SSQG_02491 and SSQG_00225 are missing, so the numbers of amino acid residues are too short to compare. In fact, blast analysis shows that SHJG_8639 is similar to Zn-dependent hydrolases (l-ascorbic acid metabolism, UlaG) of *Streptomyces corchorusii* (WP_059265188), *S. bingchenggensis* (WP_043485943) , and *Nonomuraea candida* (WP_043633066); SHJG_2828 is similar to Zn-dependent hydrolases (glyoxylase, GloB) of multiple species of *Streptomyces* (WP_037824258, WP_037629085 and others), *Streptomyces fulvoviolaceus* (WP_03061340) , and *S*. *avermitilis* (WP_010988349); SSCG_03668 is related to serine hydrolases of *Streptomyces aureus* (WP_037619730), *Actinokineospora spheciospongiae* (WP_035277635) and *Micromonospora parva* (WP_030334201); SRIM_31085 is similar to PBPs of *S. rimosus* (WP_030645517), *S. albus* (WP_060729102) and *S. yokosukanensis* (KUN09412); BN_7932 is associated with serine hydrolases of *Streptomyces venezuelae* (WP_055639726), *Streptomyces vietnamensis* (WP_041132293), and *Streptomyces antibioticus* (WP_059193915); SSQS_02491 is similar to serine hydrolases of *Streptomyces olindensis* (KDN73989), *Streptomyces acidiscabies* (WP_059045061), and *Streptomyces torulosus* (WP_055716632); and SSQG_00225 is related to PBPs of *Streptomyces chartreusis* (WP_010033556), *Streptomyces iakyrus* (WP_051814832), and *Streptomyces pactum* (WP_055421147). Therefore, all of these sequences are members of the β-lactamase superfamily. The amino acid numbers of SHJG_2828, SSCG_03668, SRIM_31085, SSQG_02491 and SSQG_00225 are too short to function as β-lactamases, and the C-terminal residues in SSCG_03303 are missing. 

**Figure 3:**
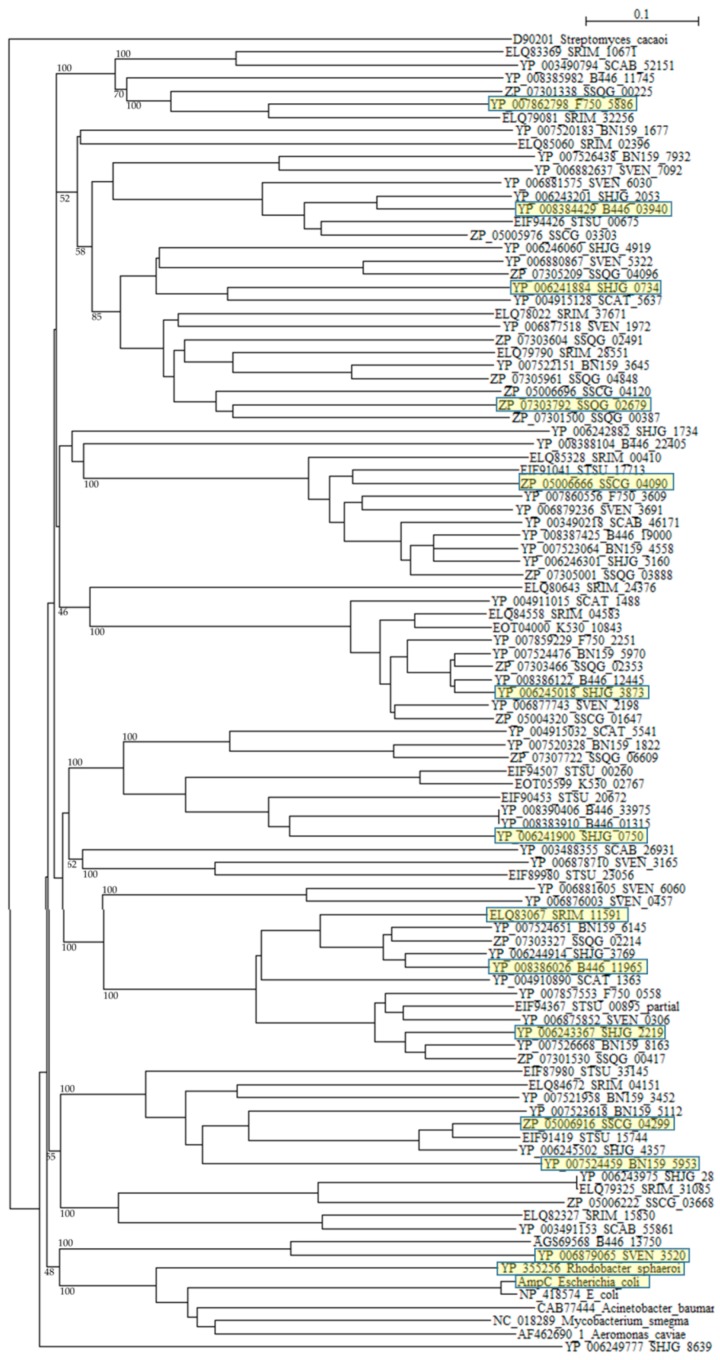
Phylogenetic tree of putative 94 class C β-lactamases on the basis of amino acid sequences. The tree was constructed by using ClustalX 2 [88] and *Streptomyces* *cacaoi* class A β-lactamase (D90201) as outgroup. β-lactamases of *Rhodobacter sphaeroides* (YP_355265), *Mycobacterium smegmatis* (NC_018289), *Acinetobacter baumannii* (CAB77444), *Aeromonas caviae* (AF462690_1), and *E. coli* (ABM69263 and NP_418574) were used as reference sequences. The sequences marked with yellow boxes were used for the calculation of molecular distances (Table S6). The bootstrap probabilities are shown at branching nodes.

A phylogenetic tree was constructed with β-lactamases of *Rhodobacter sphaeroides* (YP_355265), *Mycobacterium smegmatis* (NC_018289), *Acinetobacter baumannii* (CAB77444), *Aeromonas caviae* (AF462690_1), and *E. coli* (ABM69263 and NP_418574) as reference sequences (Figure 3). Although six reference sequences are selected from various different species [95], they form only one cluster as described previously [77,78]. On the other hand, β-lactamases of *Streptomyces* are dispersed into numerous clusters, indicating that they originate from different kinds of proteins. A molecular distance analysis using the protdist program [90] confirmed this observation (Table S6). The molecular distances between *Streptomyces* β-lactamases and that of pathogenic bacteria are very large, but those of β-lactamases of *Streptomyces* are also large with each other, so if we continue to develop and use new, newer and the newest β-lactam antibiotics at the present rate, a dreadful microbial revolution may occur near future. 

### 2.2. Penicillin-Binding Proteins (PBPs)

The bacterial cell wall peptidoglycan is a three-dimensional, covalently closed, net-like mesh called sacculus in which glycan strands are cross-linked by peptide chains. It maintains cell shape and provides mechanical strength to resist osmotic pressure [96,97]. The peptidoglycan chains are composed of alternating β-1,4-linked *N*-acetylglucosamine and *N*-acetylmuramic acid residues. The carboxyl groups of the *N*-acetylmuramic acid residues are involved in amide bond to terminal l-alanine residues of the peptide units l-alanyl-γ-d-glutamyl-l-diaminoacyl-d-alanine. The glycan chain is biosynthesized by catalysis of glycosyltransferases and the cross-linkage of the peptide chains between two adjacent glycan chains is catalyzed by transpeptidases [87,98]. The transpeptidases, also called penicillin-binding proteins (PBPs), were initially identified as their ability to bind penicillins [99,100,101]. Depending on the structure and the catalytic activity of their *N*-terminal domain, they are classified into class A, class B and class C PBPs [87,96,101,102]. Both class A and class B PBPs have the transpeptidase activity in the C-terminal region. However, the *N*-terminal domain in class A PBPs is responsible for their glycosyltransferase activity, while the glycosyltransferase domain is lacking in class B PBPs. Instead, the PBP dimer domain (Pfam03717) is present at the *N*-terminal region in class B PBPs. However the detailed functional role of the dimer domain is yet to be fully explicated. Class C PBPs are also called low molecular weight PBPs and, having the carboxypeptidase activity, are responsible for the maturation and recycling of the peptidoglycan [96], but they are not essential and are excluded from further study. There are many excellent review paper on PBPs [87,96,97,98,100,101,102], and the comprehensive review on the PBPs in *Actinobacteria* in general was already published [103], so this review describes the PBPs in *Streptomyces*, especially emphasizing their roles in self-resistance.

#### 2.2.1. Class A PBPs

**Figure 4:**
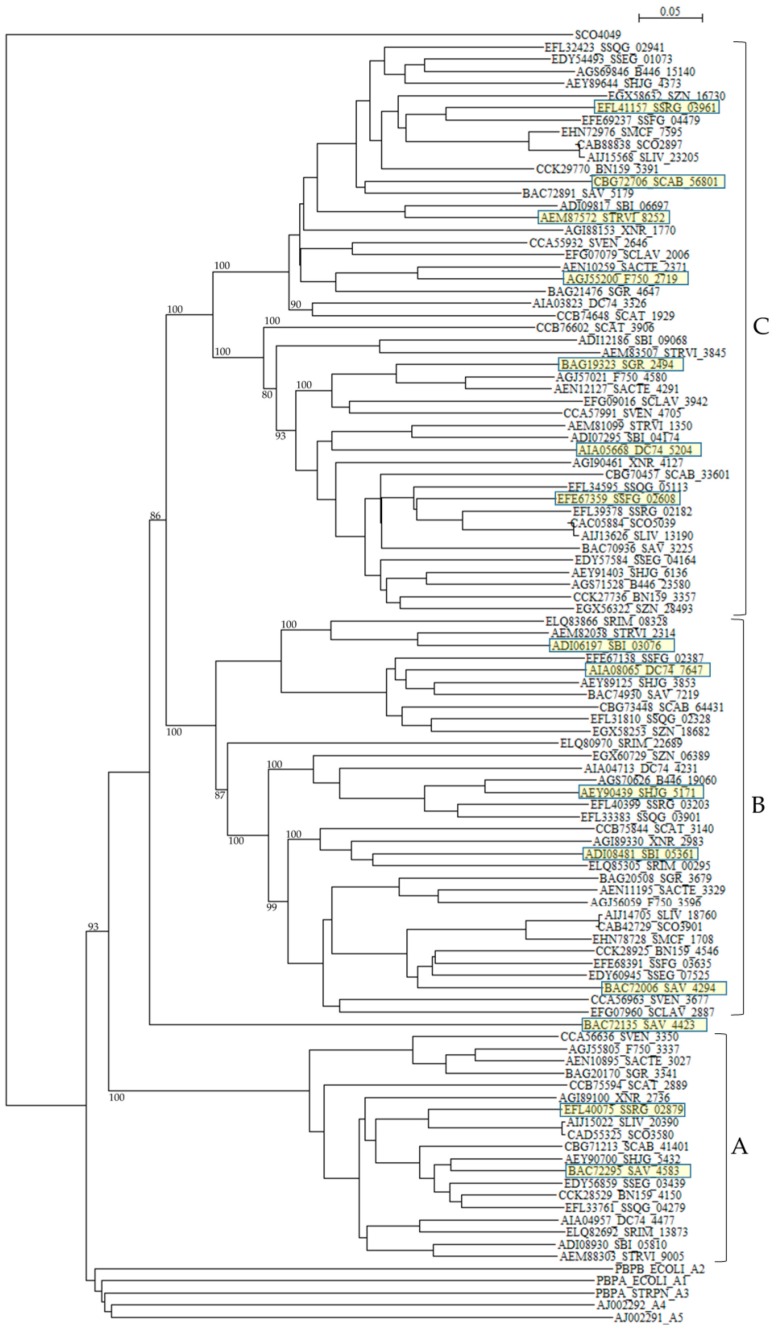
Phylogenetic tree of putative 100 class A PBPs on the basis of amino acid sequences. The tree was constructed by using ClustalX 2 [88] and *S. coelicolor* putative penicillin acylase (SCO4049, CAC32320) as outgroup. *E. coli* PBP1A (subclass A1, PBPA_ECOLI), *E. coli* PBP1B (subclass A2, PBPB_ECOLI), *S. pneumoniae* PBP1A (subclass A3, PBPA_STRPN), *S. pneumoniae* PBP2A (subclass A4, AJ002292), and *S. pneumoniae* PBP1B (subclass A5, AJ002291) were used as reference sequences. The sequences marked with yellow boxes were used for the calculation of molecular distances (Table S8). The PBPs in groups A, B, and C are corresponding to those in clusters A, B, and C in the amino acid alignment shown in Table S7. The bootstrap probabilities are shown at branching nodes.

The numbers and types of putative PBPs in 24 *Streptomyces* are summarized in Table 6. The amino acid sequences of putative 100 class A PBPs in 24 *Streptomyces* species together with a reference sequence (Q04704/CVP62105, *S. pneumoniae*) were aligned by the Muscle and T-coffee programs (Table S7). All sequences analyzed possess active site S^465^XXK^468^ residues, excluding SAV_4423, SBI_09068, STRVI_3845, SCAB_64431, SZN_18682, DC74_7647, SSQG_02328, SSFG_02387, SAV_7219, SHJG_3853, SRIM_08328, SBI_03076, and STRVI_2314. However, active site residues S^524^XN^546^, and K^716^TG^718^ are mostly conserved. In fact, blast analysis of STRVI_3845 as a query showed that glycosyltransferases of *S. iranensis* (WP_044567878), *S. hygroscopicus* (WP_060948597) and *S. bingchenggensis* (WP_014181633) are identified and, that of SSQG_02328 as a query, PBPs of *S. afghaniensis* (WP_037667886), *S. iakyrus* (WP_033306427), and *S. caeruleatus* (KUN93289) are identified, and that of SHJG_3853 as a query, PBPs of *S. antibioticus* (WP_053211715), *S. reticuli* (CUW28219), and *S. achromogenes* (WP_030604457) are identified. In addition, the glycosyltransferase and transpeptidase domains are preserved in these sequences, indicating that these sequences can function as PBPs. A phylogenetic tree was constructed by using SCO4049 (putative penicillin acylase) as outgroup (Figure 4). The class A PBPs are clearly divided to three groups: A, B and C. SAV_4423 is a lone sequence. The PBPs in these groups are obviously corresponding to those in clusters A, B and C in the amino acid alignment (Table S7).

According to the penicillin-binding cores of PBPs, class A PBPs are classified into two subclasses in Gram-negative bacteria and three subclasses in Gram-positive bacteria [104]: A1 (whose prototype is *Escherichia coli* PBP1A, PBPA_ECOLI), A2 (whose prototype is *E. coli* PBP1B, PBPB_ECOLI), A3 (whose prototype is *Streptococcus pneumoniae* 1A, PBPA_STRPN), A4 (whose prototype is *S. pneumoniae* 2A, AJ002292), and A5 (whose prototype is *S. pneumoniae* 1B, AJ002291). Interesting enough, all class A PBPs from *Streptomyces* analyzed in this paper form a completely different cluster in the phylogenetic tree from these five subclasses (Figure 4), where E values (number of alignments expected by chance) between PBPs in subclasses A1 to A5 and those in *Streptomyces* are in the range of 3.4 × 10^–16^ to 4.9 × 10^–31^, indicating low similarities. These results suggests strongly that the gene transfer and/or gene conversion occurred rarely between PBPs in *Streptomyces* and those in Gram-positive and Gram-negative bacteria. However, the molecular distances are not so large except PBP of *Helicobacter pylori* and PBPs of *Streptomyces*, indicating that these PBPs are not so different as speculated by the phylogenetic tree with each other (Table S8), and it is possible that the horizontal gene transfer of these genes may take place from *Streptomyces* to pathogenic bacteria near future.

#### 2.2.2. Class B PBPs

**Figure 5:**
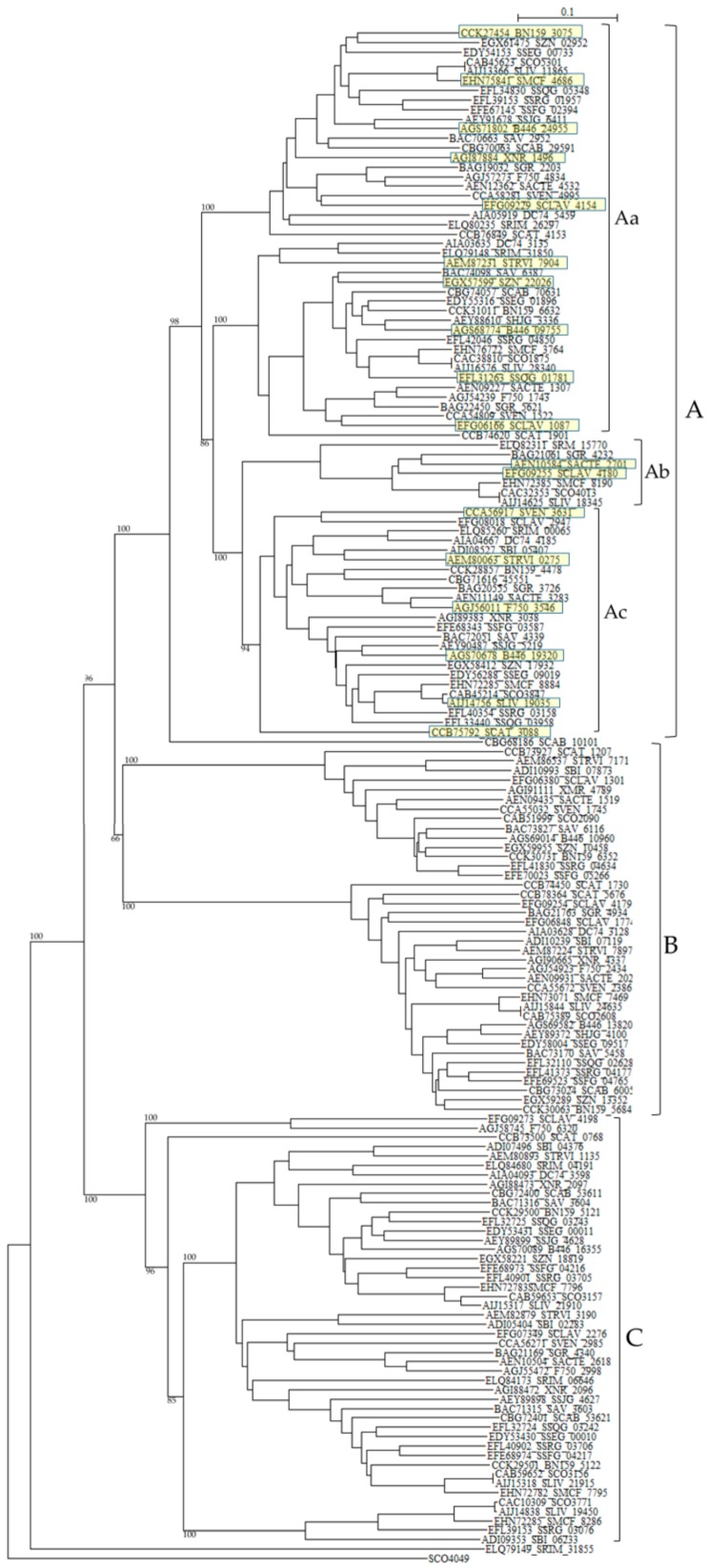
Phylogenetic tree of putative 161 class B PBPs on the basis of amino acid sequences. The tree was constructed by using ClustalX 2 [88] and *S. coelicolor* putative penicillin acylase (SCO4049, CAC32320) as outgroup. The sequences marked with yellow boxes were used for the calculation of molecular distances (Table S9). The bootstrap probabilities are shown at branching nodes. The phylogenetic tree were arbitrary divided to three groups, A, B, and C.

One hundred sixty one class B PBPs in 24 *Streptomyces* listed in Table 6 were analyzed. Intriguingly, more than half of *Streptomyces* species carry two successive class B PBPs in tandem. For example, XNR_2096 and XNR_2097 (E value is 5.4 × 10^–58^, the same hereafter), SAV_3603 and SAV_3604 (3 × 10^–68^), SCLAV_4178 and SCLAV_4180 (1.5 × 10^–11^), SMCF_7795 and SMCF_7796 (3.3 × 10^–60^), SCO3156 and SCO3157 (2.8 × 10^–47^), BN159_5121 and BN159_5122 (1.4 × 10^–62^), SSFG_04216 and SSFG_04217 (1.3 × 10^–56^), SSRG_03705 and SSRG_03706 (2.7 × 10^–62^), SHJG_4627 and SHJG_4628 (2.8 × 10^–69^), SLIV_21910 and SLIV_21915 (4.3 × 10^–58^), SCAB_53611 and SCAB_53621 (6.8 × 10^–53^), SSEG_00010 and SSEG_00011 (3.8 × 10^–63^), and SSQG_03242 and SSQG_03243 (5.5 × 10^–65^). These amino acid sequences are not only very similar to each other, but also all the sequences belong to the same cluster in a phylogenetic tree constructed by using SCO4049 as outgroup (cluster C in Figure 5). In fact, the amino-acid sequence identity and similarity of PBPs in cluster C in the phylogenetic tree are in the range of 49.2%–51.8% and 71.8%–77.8%, respectively. Moreover, the nucleotide sequences of each pair are arrayed in the same direction, indicating that they were duplicated and transferred to each other very recently. Interestingly enough, the pair of a cephamycin and clavulanic acid producer, that is, *S. clavuligerus* SCLAV_4179 and SCLAV_4180 is an exception. 

SCLAV_4179 belong to cluster B and SCLAV_4180 pertain to cluster A in the phylogenetic tree and the similarity of the amino-acid sequences is very low (E-value is 1.5 × 10^–11^). This character may be related to β-lactam production and these two PBPs seem to behave independently, because these two sequences are aligned in the reverse direction, *i.e.*, SCLAV_4180 (pbpA) acts together with the clavulanic acid biosynthetic gene cluster, while SCLAV_4179 (pbp2) goes on with SCLAV_4178 (cytochrome P450, partial start) and SCLAV_4177 (RNA polymerase) or independently [78]. Moreover, PBP SCLAV_4179 in *S. clavuligerus* is reported to have a low affinity to β-lactam antibiotics and is essential to the growth [105]. Therefore, it is assumed that PBP SCLAV_4179 is involved in the self-resistance. SCLAV_4198 (class BPBP, pcbR) is located in the border between cephamycin and clavulanic acid biosynthetic gene clusters but behaves independently from these clusters, considering the gene direction. In *S. cattleya* which produces only cephamycin but not clavulanic acid, genes corresponding to SCLAV_4180 and SCLAV_4198 are deleted, but a gene (SCAT_5676) corresponding to SCLAV_4179 and responsible for self-resistance is retained [78]. This fact supports the above ideas (Figure S2). 

Related to self-resistance, Ogawara and Horikawa reported nearly 40 years ago that β-lactam-producing *Streptomyces* species such as *S. clavuligerus* possessed PBPs of very low affinity to benzylpenicillin [106]. Later, two PBPs in *S. clavuligerus*, that is, SCLAV_4179 and SCLAV_4198 were reported to have low affinity to penicillins [105,107]. A mutant disrupted in SCLAV_4198 gene exhibited a significant decrease in its resistance to benzylpenicillin and cephalosporins [107]. Moreover, a probe containing SCLAV_4198 hybridized to genomic DNAs from β-lactam producers, *S. jumonjinensis* NRRL 5741, *S. griseus* NRRL 3851 and *S. lipmanii* NRRL 3584, suggesting that SCLAV_4198-like sequences and SCLAV_4198-mediated resistance mechanisms are likely to be present in these β-lactam-producing species. Likewise, most *Streptomyces* species have two PBPs in SCLAV_4198-belonging cluster C in the tree.

These characters are moderately similar with each other: SRIM_04191 and SRIM_06646 (1.4 × 10^–59^), SCLAV_4198 and SCLAV_2276 (8.1 × 10^–27^), F750_6320 and F750_2998 (5.6 × 10^–29^), STRVI_1135 and STRVI_3190 (1.2 × 10^–61^), and SBI_04376 and SBI_06233 (7.7 × 10^–36^) are examples. Together with the fact that tandem two genes in most *Streptomyces* species are associated with cluster C as described above, these results clearly indicate that most *Streptomyces* species are firmly defended by the presence of two low-affinity PBPs. Moreover, cluster B PBPs, to which SCLAV_4179 belong, reconfirm the self-resistance [103], that is, two pairs of low affinity PBPs (e.g., SCLAV_4198 and SCLAV_2276 in cluster C in Figure 5, and SCLAV_4179 and SCLAV_1774 in cluster B in Figure 5) guard *Streptomyces* from the attack of β-lactam antibiotics. 

## 3. PASTA Domains: Connector between Penicillin-Binding Proteins and Protein Kinases

Protein phosphorylation was first described as a major regulatory mechanism in eukaryotes [108,109]. Later, the regulation by phosphorylation with serine/threonine protein kinases (STPKs) in particular has been expanded to prokaryotes [110,111,112,113]. While investigating the *S. coelicolor* homolog of PknB, a STPK, Yeats *et al.* identified a definitive domain in its C-terminus. This domain is also found in C-terminus of high molecular weight PBPs, so that it is termed as the PASTA domain (penicillin-binding protein and serine/threonine kinase-associated domain) [114]. PASTA domains are not found in eukaryotes and are restricted to firmicutes and *Actinobacteria*. Interestingly, some PASTA domains in PBPs such as PBP2x of *S. pneumoniae* bind peptidoglycan and β-lactam antibiotics [115], but others such as PonA2 of *M. tuberculosis* do not [116], indicating that PASTA domains in PBPs operate in a species-specific manner. On the other hand, PASTA domains in STPKs bind peptidoglycan and β-lactam antibiotics, and STPKs with PASTA domains are reported to play important roles in the regulation of bacterial cell division, and morphogenesis [111,112,114,117,118]. Previously, I described the distribution of PASTA domains in *Actinobacteria* [119], so in this review I discuss the interaction of PASTA domains in PBPs and STPK from the point of view of self-resistance. 

In sharp contrast to other bacteria such as *B. subtilis, Clostridium perfringens and S. pneumonia*, PBPs with PASTA domains are detected only in class A PBPs but not in class B PBPs of *Actinobacteria*. In addition, none of *Streptomyces* species has PBPs with PASTA domain. Class A PBPs have both transglycosylase and transpeptidase domains, whereas class B PBPs have only transpeptidase domain. Therefore, it is interesting to know the interaction between transglycosylase and PASTA domains in *Actinobacteria*. However, it is not interpreted yet. Related to this fact, one of class B PBPs without PASTA domain and a STPK with four PASTA domains are located in adjacent position in most *Streptomyces* species (Table 6). For example, DC74_4185 and DC74_4186, XNR_3038 and XNR_3037, SAV_4339 and SAV_4338, SBI_05407 and SBI_05406, SCO3847 and SCO3848, and others. These PBPs belong to cluster Ac in a phylogenetic tree (Figure 5). The genomic structures in PBP-STPK regions of *S. coelicolor*, *S. clavuligerus*, and *S. griseus* are very similar and suggest that STPK, PBP and FtsW/RodA family protein form an operon (Table 7). In *S. coelicolor*, this family of PBP (*i.e.*, SCO3847) is reported to form the *Streptomyces* spore wall synthesizing complex (SSSC) [120,121]. Although they are supposed to be transported to peptidoglycan biosynthesis machinery through the PASTA domains of STPK (*i.e.*, SCO3848) and be involved in the peptidoglycan biosynthesis and/or morphogenesis coupled with PBPs and FtsW/RodA family proteins, Jones *et al.* reported that SCO3848 (PknB) regulates the timing of development and TCA cycle favoring antibiotic production together with two forkhead-associated proteins (FHA, SCO3843 and SCO3844) [122]. In addition, SCO3848 (PknB) are supposed to play coordinately with the protein phosphatase (*i.e.*, SCO3845) inside the operon. It is very interesting to know how self-resistance is implicated in this reaction, because the identity and the similarity of PBPs of this group (cluster Ac in Figure 5) to SCLAV_4180 are in the range of 40%–91%, and 70%–95%, respectively and those of SCLAV_2947 (a low affinity PBP) and SCLAV_4180 are 53.3% and 80.2% in 497 amino acid residues, respectively (E value is 1.7 × 10^–102^). The indication that clusters Aa, Ab, and Ac PBPs in Figure 5 are close together with each other is confirmed by the molecular distance determination (Table S9). Cluster A PBPs in Figure 5 are also closely related with PBPs of pathogenic bacteria (Table S9). Unfortunately, however, the role of cluster A PBPs in self-resistance remains to be elucidated.

## 4. Relationship between β-Lactam Biosynthetic Gene, β-Lactamase and PBP: Additional Comments

The relationship between β-lactam biosynthetic gene clusters, β-lactamases, and PBPs of *S. clavuligerus* and *S. cattleya* was shown in Figure S1 [33,123,124,125,126,127]. The genetic organization of clavulanic acid biosynthetic gene cluster of *S. clavuligerus* was published [93,128]. Comparison of cephamycin gene clusters in *S. clavuligerus* and *S. cattleya* indicates clearly that clavulanic acid gene cluster from SCLAV_4180 to SCLAC_4198 in *S. clavuligerus* was inserted between SCAT_5676 and SCAT_5678 of *S. cattleya* (Figure S2). SCAT_5677 is missing. Interestingly, both sides of the clavulanic acid gene cluster are occupied by PBPs (SCLAV_4180 and SCLAV_4198), indicating that these PBPs behave together with clavulanic acid/cephamycin gene cluster and are involved in the self-resistance of *S. clavuligerus*. Similar gene constructs for cephamycin biosynthesis are observed in *Nocardia lactamdurans*, *Lysobacter lactamgenus*, *Penicillium chrysogenum*, and *Acremonium chrysogenum* [129,130,131,132]. However, β-lactamase and PBP genes can be detected neither in β-lactam biosynthetic gene clusters nor in whole genomes of *P*. *chrysogenum*, and *A*. *chrysogenum*. On the other hand, the organizations of clavulanic acid gene clusters of three *Streptomyces species*, *S. clavuligerus*, *S. flavogirseus*, and *S. viridis* are also similar with each other. Unfortunately, however, relationship between β-lactam biosynthesis, β-lactamases, and PBPs remains to be clarified, although β-lactamase was reported to be expressed during the active growth phase, prior to the formation of β-lactam antibiotics [129]. Recently, an interesting paper on taxonomy, physiology, and natural products of *Actinobacteria* was published [133].

## 5. Conclusions

Although antibiotics still play a key role for the prevention of microbial infections as remaining treasures from the twentieth century, antibiotic resistance is prevailing and putting us in a critical situation. Furthermore, it is said that the post-antibiotic era is coming soon [134]. As described in this paper, the β-lactamases in antibiotic-producing *Streptomyces* are diverse in their characteristics, and the PBPs are multiplexed in their guard systems. In addition, as antibiotic resistance mechanisms are supposed to originate and evolve in their producing microbes, and be transferred to pathogenic bacteria by transformation, transduction, transfection and/or conjugation, the public health crisis will be getting worse and worse. β-Lactamases and PBPs are two major resistance mechanisms in pathogenic bacteria against β-lactam antibiotics, the most frequently used antibiotics for infectious diseases at the present time. Moreover, from about 40 years ago, the rate of discovery of new antibiotics has declined rapidly and many large pharmaceutical companies have abandoned research and development on antibiotics [135]. To avoid this situation, therefore, it is urgently needed to make the public and the government recognize the situation. In addition, new antibiotics should be screened by using various technologies such as activation of cryptic gene clusters for antibiotic biosynthesis, metagenomics mining, combinatorial biosynthesis, target-directed computer-aided chemical synthesis, systems biology, genetic manipulation, omics, and so on [136,137,138,139,140,141]. Furthermore, combination therapies with monoclonal antibodies and vaccines are also required. When these requirements are attained, “New Golden Age” is possible [141]. However, it is worth mentioning again that the resistance mechanisms in *Streptomyces* species are very hard to deal with as described in this paper.

## Figures and Tables

**Table 1:** Classification of β-lactamases in 12 *Streptomyces* species.

Bacteria	Prefix	Class A	Class B	Class C	Class D
*Streptomyces albulus CCRC11814*	K530	01315, 35835 *	18626, 25018, 25240	02767, 10843, 40281	none
*Streptomyces cattleya NRRL805*	SCAT	0807, 1418, 4581, 5692	0258, 1598, 4145, 4517, 4822	1363, 1488, 5541, 5637	none
*Streptomyces clavuligerus ATCC27064*	SSCG	00130, 00160, 01467	00563, 00791, 02181, 04638, 05032, 05130, 05295, 05571	01647, 03303, 03668, 04090, 04120, 04299	none
*Streptomyces collinus Tu365*	B446	02285, 33010	02000, 02210, 05525, 13210, 29385, 33085, 33290	01315, 03940, 11745, 11965, 12445, 13750, 19000, 22405, 33975	none
*Streptomyces davawensis JCM4913*	BN159	5790	0265, 0293, 4295, 5380, 5740, 6071, 7079,7978, 8291	1677, 1822, 3452, 3645, 4558, 5112, 5953, 5970, 6145, 7932, 8163	none
*Streptomyces hygroscopicus* subsp. *jinggangensis* 5008	SHJG	8264, 8266	0444, 1156, 1422, 2523, 3996, 4046, 8335	0734, 0750, 1734, 2053, 2219, 2828, 3769, 3873, 4357, 4919, 5160, 8639	none
*Streptomyces rimosus subsp. rimosus ATCC10970*	SRIM	07618, 10531	08043, 16115, 16845, 19739, 21549, 23716, 26422	00410, 02396, 04151, 04583, 10671, 11591, 15850, 24376, 28551, 31085, 32256, 37671	none
*Streptomyces scabiei 87.22*	SCAB	38731	25181	26931, 46171, 52151, 55861	none
*Streptomyces sp. PAMC26508*	F750	2387, 6336, 6823	0075, 2374	0558, 2251, 3609, 5886	none
*Streptomyces tsukubaensis NRRL18488*	STSU	15167, 15177, 32330	07338, 08188, 24671, 31620	00260, 00675, 00895, 15744, 17713, 20672, 23056, 33145,	none
*Streptomyces venezuelae ATCC 10712*	SVEN	0089	0710, 1591, 2335, 2660, 7291	0306, 0457, 1972, 2198, 3165, 3520, 3691, 5322, 6030, 6060, 7092	none
*Streptomyces viridochromogenes DSM 40736*	SSQG	01127	02569, 02955, 05776, 05938, 06409	00225, 00387, 00417, 02214, 02353, 02491, 02679, 03888, 04096, 04848, 06609	none

*: Prefix K530-RS; Accession Number: WP_020929156 and WP_037636700, respectively.

**Table 2:** Presence or absence of active site residues in class A β-lactamases.

β-Lactamase	SXXK^73^	SDN^132^	RW/YE^166^	D^179^	KT/SG^236^
YP_004910945_SCAT_1418	X	X	X	○	X
YP_004914072_SCAT_4581	X	X	X	○	X
ZP_05002833_SSCG_00160	○	○	△	○	○
YP_007863245_F750_6336	○	○	△	○	○
YP_006249403_SHJG_8264	○	○	X	E	○
YP_008384100_B446_02285	○	○	○	X	○
YP_008390217_B446_33010	○	○	○	X	○
EIF91547_STSU_15177	○	○	△	X	○
YP_004910346_SCAT_0807	○	○	△	X	○
YP_004915182_SCAT_5692	○	○	HYE	○	○
ZP_05002803_SSCG_00130	○	○	QYE	○	○
WP_037636700_K530_RS35835	○	○	○	○	○
EIF91545_STSU_15167	○	○	○	○	○
ELQ83985_SRIM_07318	X	X	○	○	○
WP_020929156_K530_RS01315	○	○	○	○	○
YP_006249405_SHJG_8266	○	○	○	○	○
ELQ83341_SRIM_10531	○	○	○	○	○
YP_003489505_SCAB_38731	○	○	○	○	○
YP_007524296_BN159_5790	○	○	○	○	○
ZP_05003887_SSCG_01467	○	○	○	○	○
YP_006875636_SVEN_0089	○	○	○	○	○
ZP_07302240_SSQG_01127	○	○	○	○	○
YP_007859362_F750_2387	X	X	○	E	X
EIF88144_STSU_32330	○	○	X	X	○
YP_007863731_F750_6823	○	○	○	○	○

○: presence; X: absence; △: probable; other symbols are for amino acid residues.

**Table 3:** The list of active site residues discussed in this paper.

Class A β-lactamases	S^70^XXK^73^	S^130^DN^132^	R^164^W/YE^166^	D^179^	K^234^T/SG^236^	
Class B β-lactamases:	H^116^XHXD^120^	G^123^	H^196^	H^263^		
Class C β-lactamases	S^64^XXK^67^	Y^150^XN^152^			K^315^TG^317^S/A^318^	
Class D β-lactamases	S^67^TFK^70^	S^115^XV	Y^142^G	W^154^	K^205^TG	W^221^XXG
Class A PBPs	S^465^XXK^468^	S^524^XN^526^			K^716^TG^718^	
Class B PBPs:	S^307^XXK^310^	S^356^XN^358^			S^356^XN^358^	

The amino acid numbering follows Ambler [65] for classes A and D, Galleni *et al.* [85] for class B, and Lobkovsky *et al.* [86] for class C β-lactamases, and Goffin and Ghuysen [87] for PBPs.

**Table 4:** Presence or absence of active site residues in class B β-lactamases.

β-Lactamase	HXHXD^120^	G^123^	H^196^	H^263^
EOT02442_K530_18626	○	D	X	X
YP_006878205_SVEN_2660	○	D	X	X
YP_007523886_BN159_5380	○	D	X	X
ZP_07304068_SSQG_02955	○	D	X	X
ZP_05007878_SSCG_05130	△	○	○	○
YP_006249473_SHJG_8335	△	○	○	○
YP_004913644_SCAT_4145	△	○	○	○
ELQ82101_SRIM_16845	△	○	○	○
ZP_07307522_SSQG_06409	○	E	X	R
**AAA22276_Bacillus_cereus**	○	○	○	○
YP_008384085_B446_02210	○	○	X	○
YP_008390232_B446_33085	○	○	X	○
ZP_05004854_SSCG_02181	○	○	○	○
YP_004909803_SCAT_0258	○	○	○	○
ELQ82279_SRIM_16115	○	○	○	○
ZP_05007361_SSCG_04638	○	L	○	R
YP_007524577_BN159_6071	○	L	○	○
YP_007857070_F750_0075	○	Y	○	○
ZP_05003465_SSCG_00791	○	○	E	○
ZP_05007967_SSCG_05295	○	○	○	○
ELQ83811_SRIM_08043	○	○	○	○
YP_004911125_SCAT_1598	○	○	○	○
ZP_07303682_SSQG_02569	○	○	○	○
YP_007524246_BN159_5740	○	○	○	○
YP_008386271_B446_13210	○	○	○	○
YP_006245191_SHJG_4046	○	○	○	○
YP_007859349_F750_2374	○	○	○	○
YP_006877880_SVEN_2335	○	○	○	○
ZP_05007774_SSCG_05032	○	○	○	○
EIF89665_STSU_24671	○	○	○	○
YP_007518799_BN159_0293	○	N	○	○
YP_006242571_SHJG_1422	○	W	○	○
ELQ80751_SRIM_23716	○	W	○	○
YP_006242306_SHJG_1156	○	W	○	X
ELQ81190_SRIM_21549	○	W	○	T
YP_006241594_SHJG_0444	○	W	○	D
YP_006245141_SHJG_3996	○	W	○	X
YP_008384043_B446_02000	○	S	○	○
YP_008389494_B446_29385	○	○	G	A
ZP_07307051_SSQG_05938	○	○	G	A
YP_004914313_SCAT_4822	○	○	G	A
YP_004914015_SCAT_4517	○	○	G	T
YP_003488184_SCAB_25181	○	A	G	S
ZP_07306889_SSQG_05776	○	○	G	S
ELQ81507_SRIM_19739	○	○	G	S
ZP_05008219_SSCG_05571	○	○	G	S
EIF92931_STSU_08188	○	○	G	S
YP_007526484_BN159_7978	○	○	○	○
YP_007526796_BN159_8291	○	○	○	○
EOT01146_K530_25240	○	○	X	○
EIF93163_STSU_07338	○	○	X	○
ELQ80212_SRIM_26422	○	S	○	○
YP_008384742_B446_05525	○	○	○	○
YP_006243671_SHJG_2523	○	○	○	○
EIF88273_STSU_31620	○	A	○	○
YP_006876256_SVEN_0710	○	A	○	○

○: presence; X: absence; △: probable; other symbols are for amino acid residues.

**Table 5:** Presence or absence of active site residues in class C β-lactamases.

β-Lactamase	SXXK^70^	YXN^152^	KTG^317^
YP_006249777_SHJG_8639	○	X	X
YP_006243975_SHJG_2828	X	X	○
YP_006242882_SHJG_1734	○	○	X
AGS69568_B446_13750	○	○	○
YP_006879065_SVEN_3520	○	○	HTG
ZP_05006222_SSCG_03668	X	X	X
AmpC_Escherichia_coli	○	○	○
ELQ82327_SRIM_15850	○	YSS	HDG
ELQ79325_SRIM_31085	X	X	X
YP_003491153_SCAB_55861	○	YSG	HDG
EIF89980_STSU_23056_391	○	○	○
YP_006878710_SVEN_3165	○	X	RAG
YP_007520183_BN159_1677	○	○	HTG
YP_007526438_BN159_7932	X	YSH	X
YP_006882637_SVEN_7092	△	YSH	X
YP_003488355_SCAB_26931	○	○	HDG
ELQ85060_SRIM_02396	○	○	○
YP_006880867_SVEN_5322	○	○	○
ZP_07305209_SSQG_04096	○	○	○
YP_004915128_SCAT_5637	○	YNG	○
YP_006241884_SHJG_0734	○	YNG	○
YP_006881575_SVEN_6030	○	○	HNG
YP_008384429_B446_03940	○	○	HNG
YP_006243201_SHJG_2053	○	○	HNG
EIF94426_STSU_00675	○	○	HNG
ZP_05005976_SSCG_03303	○	○	X
YP_006246060_SHJG_4919	○	YRG	○
YP_006877518_SVEN_1972	○	○	HGG
ELQ78022_SRIM_37671	○	○	HGG
YP_008388104_B446_22405	○	CSN	RSG
ZP_07303604_SSQG_02491	X	○	HDG
ELQ79790_SRIM_28551	○	○	HNG
YP_007522151_BN159_3645	○	○	HSG
ZP_07305961_SSQG_04848	○	○	HNG
ZP_05006696_SSCG_04120	○	○	HSG
ZP_07301500_SSQG_00387	○	○	KSG
ZP_07303792_SSQG_02679	○	○	HGG
EIF87980_STSU_33145	○	YHA	X
YP_007521958_BN159_3452	○	YHS	X
YP_007524459_BN159_5953	○	YHA	○
ELQ84672_SRIM_04151	○	YHA	HGG
YP_007523618_BN159_5112	○	YHA	HPG/RGG
YP_006245502_SHJG_4357	○	YHG	HTG/RGG
ZP_05006916_SSCG_04299	○	YHG	HTG/RGG
EIF91419_STSU_15744	○	YHG	HTG/RGG
YP_004915032_SCAT_5541	○	YSV	X
YP_007520328_BN159_1822	○	YSV	X
ZP_07307722_SSQG_06609	○	YSV	○
EOT05599_K530_02767	○	YNV	X
EIF94507_STSU_00260	○	YHV	X
EIF90453_STSU_20672	○	YNT	○
YP_008383910_B446_01315	○	YDT	RVG
YP_008390406_B446_33975	○	YDT	RVG
YP_006241900_SHJG_0750	○	YGT	RYG
ELQ85328_SRIM_00410	○	○	HSG
ZP_05006666_SSCG_04090	○	○	HSG
EIF91041_STSU_17713	○	○	HTG
YP_003490218_SCAB_46171	○	○	HGG
ZP_07305001_SSQG_03888	○	○	HTG
YP_006246301_SHJG_5160	○	○	HSG
YP_007523064_BN159_4558	○	○	HSG
YP_008387425_B446_19000	○	○	HSG
YP_006879236_SVEN_3691	○	○	HTG
YP_007860556_F750_3609	○	○	HSG
YP_003490794_SCAB_52151	○	○	○
ELQ83369_SRIM_10671	○	○	HNG
YP_008385982_B446_11745	○	○	HDG
ZP_07301338_SSQG_00225	X	○	HSG
ELQ79081_SRIM_32256	○	○	HSG
YP_007862798_F750_5886	○	○	HSG
ELQ80643_SRIM_24376	○	○	X
YP_006876003_SVEN_0457	○	YTD	HFG
YP_006881605_SVEN_6060	○	YTD	HYG
YP_004911015_SCAT_1488	○	○	HFG
ZP_05004320_SSCG_01647	○	○	HFG
YP_006877743_SVEN_2198	○	○	HFG
EOT04000_K530_10843	○	○	HFG
ELQ84558_SRIM_04583	○	○	HFG
YP_007859229_F750_2251	○	○	HFG
YP_007524476_BN159_5970	○	○	HFG
ZP_07303466_SSQG_02353	○	○	HFG
YP_006245018_SHJG_3873	○	○	HFG
YP_008386122_B446_12445	○	○	HFG
YP_007857553_F750_0558	○	YSD	HTG
YP_006243367_SHJG_2219	○	YSD	HTG
YP_007526668_BN159_8163	○	YSD	HTG
ZP_07301530_SSQG_00417	○	YSD	HTG
EIF94367_STSU_00895	○	YSD	HTG
YP_006875852_SVEN_0306	○	YSD	HTG
YP_004910890_SCAT_1363	○	YSD	HTG
ELQ83067_SRIM_11591	○	YSD	HTG
ZP_07303327_SSQG_02214	○	YSD	HTG
YP_007524651_BN159_6145	○	YSD	HTG
YP_008386026_B446_11965	○	YSD	HTG
YP_006244914_SHJG_3769	○	YSD	HTG

○: presence; X: absence; △: probable; other symbols are for amino acid residues.

**Table 6:** The numbers and types of putative PBP and protein kinase genes.

Bacteria	Prefix	Genome Size (Mb)	Class A PBP	Class B PBP	PBP with PASTA Domain *	No of Protein Kinase	Protein Kinase with PASTA Domain *
*Streptomyces albulus* NK660	DC74	9.37	3326, 4231, 4477, 5204, 7647	3128, 3135, 3598, 4185, 5459	none	40	2595(4), 4186(4)
*Streptomyces albus* J1074	XNR	6.84	1770, 2736, 2983, 4127	1496, 2096, 2097, 3038, 4337, 4789	none	25	3037(4), 3064(1), 4768(4)
*Streptomyces avermitilis* MA-4680	SAV	9.12	3225, 4294, 4423, 4583, 5179, 7219	2952, 3603, 3604, 4339, 5458, 6116, 6387	none	33	4338(4), 4371(1), 6092(4)
*Streptomyces bingchenggensis BCW-1*	SBI	11.94	03076, 04174, 05361, 05810, 06697, 09068	02283, 04376, 05407, 06233, 07119, 07873	none	67	05406(4), 07851(4)
*Streptomyces cattleya N*RRL8057	SCAT	8.09	1929, 2889, 3140, 3906	0768, 1207, 1730, 1901, 3088, 4153, 5676	none	18	1232(4), 3053(1), 3089(4)
*Streptomyces clavuligerus ATCC27064*	SCLAV	8.56	2006, 2887, 3942	1087, 1301, 1774, 2276, 2947, 4154, 4179, 4180, 4198	none	33	1326(4), 2946(4), 2991(1)
*Streptomyces coelicoflavus* ZG0656	SMCF	8.48	1708, 7595	3764, 4686, 7469, 7795, 7796, 8190, 8286, 8884	none	22	6490 (4), 8300 (1),8885 (4)
*Streptomyces coelicolor* A3(2)	SCO	9.05	2897, 3580, 3901, 5039	1875, 2090, 2608, 3156, 3157, 3771, 3847, 4013, 5301	none	37	2110(4), 3821(1), 3848(4)
*Streptomyces collinus Tu365*	B446	8.38	15140, 19060, 23580	09755, 10960, 13820, 16355, 19320, 24955	none	39	11080(4), 19315(4), 19450(1)
*Streptomyces davawensis* JCM4913	BN159	9.56	3357, 4150, 4546, 5391	3075, 4478, 5121, 5122, 5684, 6352, 6632	none	35	4396(1), 4479(4), 6328(4)
*Streptomyces ghanaensis* ATCC 14672	SSFG	8.51	02387, 02608, 03635, 04479	02394, 03587, 04216, 04217, 04765, 05266	none	35	03552(1), 03588(4)
*Streptomyces griseoflavus* Tu4000	SSRG	8.05	02182, 02879, 03203, 03961	01957, 03076, 03158, 03705, 03706, 04177, 04634, 04850	none	35	03115(1), 03159(4), 04610(4)
*Streptomyces griseus* subsp. *griseus* NBRC 13350	SGR	8.55	2494, 3341, 3679, 4647	2203, 3726, 4232, 4340, 4934, 5621	none	30	3725(4), 5391(4)
*Streptomyces hygroscopicus* subsp. *jinggangensis* 5008	SHJG	10.38	3853, 4373, 5171, 5432, 6136	3336, 4100, 4627, 4628, 5219, 6411	none	43	3594(4), 5218(4), 5252(1)
*Streptomyces lividans* TK24	SLIV	8.35	13190, 18760, 20390, 23205	11865, 18345, 19035, 19450, 21910, 21915, 24635, 28340	none	35	19030(4), 19175(1), 27155(4)
*Streptomyces rimosus* subsp. *rimosus* ATCC10970	SRIM	9.5	00295, 08328, 13873, 22689	00065, 04191, 06646, 15770, 26297, 31850, 31885	none	40	00070(4), 07563(1), 24996(4)
*Streptomyces scabiei* 87.22	SCAB	10.15	33601, 41401, 56801, 64431	10101, 29591, 45551, 53611, 53621, 60051, 70631	none	43	0931(1), 45201(1), 67711(4)
*Streptomyces* sp. PAMC26508	F750	7.63	2719, 3337, 3596, 4580	1743, 2434, 2998, 3546, 4834, 6320	none	27	1975(4), 3512(1), 3547(4)
*Streptomyces* sp. SirexAA-E	SACTE	7.41	2371, 3027, 3329, 4291	1307, 1519, 2029, 2618, 2701, 3283, 4532	none	32	1542(4), 3284(3)
*Streptomyces sviceus* ATCC 29083	SSEG	9.31	01073, 07525, 03439, 04164	00010, 00011, 00733, 01896, 09019, 09517,	none	40	02705(4), 06024(4)
*Streptomyces venezuelae* ATCC 10712	SVEN	8.23	2646, 3350, 3677, 4705	1522, 1745, 2386, 2985, 3631, 4995	none	37	1769(4), 3592(1), 3632(4)
*Streptomyces violaceusniger* Tu4113	STRVI	11.14	1350, 2314, 3845, 8252, 9005	0275, 1135, 3190, 7171, 7897, 7904	none	38	0274(4), 7194(4)
*Streptomyces viridochromogenes* DSM 40736	SSQG	8.65	02328, 02941, 03901, 04279, 05113	01781, 02628, 03242, 03243, 03958, 05348	none	37	02054(4), 03956(4), 03996(1)
*Streptomyces zinciresistens* K42	SZN	8.22	06389, 16730, 18682, 28493	02952, 10458, 13352, 17932, 18819, 22026	none	39	08009(4), 17937(4), 21616(1)

* The number in parenthesis is the numbers of PASTA domains.

**Table 7:** Comparison of genomic structures in PBP-STPK region of three *Streptomyces* species.

Gene No.	Protein Name	No. of aa	Gene No.	Protein Name	No. of aa	Gene No.	Protein Name	No. of aa
SCO3844	hypothetical protein	172aa+ *	SCLAV_2950	hypothetical protein	132aa−	SGR_3729	hypothetical protein	173aa−
SCO3845	protein phosphatase	515aa+	SCLAV_2949	protein phosphatase	507aa−	SGR_3728	protein phosphatase	499aa−
SCO3846	FtsW/RodA family protein	479aa+	SCLAV_2948	FtsW/RodA family protein	470aa−	SGR_3727	FtsW/RodA family protein	466aa−
SCO3847	PBP	490aa+	SCLAV_2947	PBP	484aa−	SGR_3726	PBP	485aa−
SCO3848	STPK	673aa+	SCLAV_2946	STPK	682aa−	SGR_3725	STPK	664aa−
SCO3849	hypothetical protein	253aa−	SCLAV_2945	hypothetical protein	231aa+	SGR_3724	hypothetical protein	245aa+
SCO3850	hypothetical protein	352aa−	SCLAV_2944	hypothetical protein	461aa+	SGR_3723	hypothetical protein	474aa+
SCO3851	glutamine amidotransferase	212aa−	SCLAV_2943	glutamine amidotransferase	212aa+	SGR_3722	glutamine amidotransferase	212aa+
SCO3852	hypothetical protein	69aa−	SCLAV_2942	hypothetical protein	68aa+	SGR_3721	hypothetical protein	62aa+

*: +: plus strand, −: minus strand.

## Supplementary Materials

Supplementary materials can be accessed at: http://www.mdpi.com/1420-3049/21/5/605/s1.
